# Isolated ACTH deficiency during single-agent pembrolizumab for squamous cell lung carcinoma: a case report

**DOI:** 10.1186/s40842-019-0092-9

**Published:** 2020-01-06

**Authors:** Sho Tanaka, Masaru Kushimoto, Tsukasa Nishizawa, Masahiro Takubo, Kazutaka Mitsuke, Jin Ikeda, Midori Fujishiro, Katsuhiko Ogawa, Ichiro Tsujino, Yutaka Suzuki, Masanori Abe

**Affiliations:** 1 Division of Nephrology, Hypertension and Endocrinology, Department of Internal Medicine, Nihon University School of Medicine, 30-1 Kamicho, Oyaguchi, Itabashi-ku, Tokyo, 173-8610 Japan; 2 Division of General Medicine, Department of Medicine, Nihon University School of Medicine, Tokyo, Japan; 3 Division of Respiratory Medicine, Department of Internal Medicine, Nihon University School of Medicine, Tokyo, Japan; 4 Division of Diabetes and Metabolic Diseases, Department of Internal Medicine, Nihon University School of Medicine, Tokyo, Japan; 5 Division of Neurology, Department of Medicine, Nihon University School of Medicine, Tokyo, Japan

**Keywords:** Adrenal insufficiency, Adrenocorticotropic hormone deficiency, Immunotherapy, Pembrolizumab, Programmed cell death 1

## Abstract

**Background:**

The programmed cell death 1 (PD-1) inhibitor pembrolizumab is a promising agent for treatment of several different malignancies, but as with all immunotherapy there is a potential risk of immune-related adverse events. Adrenocorticotropic hormone (ACTH) deficiency and hypophysitis have been reported in patients treated with a different PD-1 inhibitor, nivolumab. However, clinical characteristics of these side effects associated with pembrolizumab have yet to be described in detail.

**Case presentation:**

An 85-year-old Japanese woman was diagnosed with advanced squamous cell lung cancer. The patient was treated with 200 mg pembrolizumab every three weeks as first-line therapy. Routine examination including thyroid function, complete blood count, serum cortisol and sodium levels before each pembrolizumab infusion had shown no significant changes up to the eighth cycle. However, 8 days after the eighth cycle of single-agent pembrolizumab, she presented with rapidly worsening general fatigue and appetite loss over two days. Laboratory data revealed a low serum cortisol level (0.92 μg/dL) with inappropriately low ACTH (8.3 pg/mL), hyponatremia (122 mmol/L) and hypoglycemia (68 mg/dL). Standard-dose short ACTH testing showed an unsatisfactory cortisol response, indicating adrenal insufficiency. Pituitary magnetic resonance imaging showed diffuse substantial gadolinium enhancement, T2 hyperintensity, loss of pituitary bright spot, but no pituitary enlargement. Serum cortisol and ACTH levels were low throughout the day, and urinary free cortisol excretion fell below the lower normal limit. There was no ACTH and cortisol response in the corticotropin-releasing hormone test, despite significant responses of other anterior pituitary hormones to their corresponding challenge tests. Thus, isolated ACTH deficiency was diagnosed, and hypophysitis was suspected as the etiology. After administration of 15 mg/day hydrocortisone, the patient’s debilitation, hyponatremia, and hypoglycemia swiftly disappeared.

**Conclusion:**

This is a case of isolated ACTH deficiency possibly due to hypophysitis in a patient with advanced lung cancer, in whom recent routine examinations had shown unremarkable results. We therefore conclude that isolated ACTH deficiency can suddenly arise during pembrolizumab monotherapy, albeit probably only rarely. Caution should be exercised to make sure that adrenal insufficiency is recognized immediately in order to achieve swift recovery by steroid replacement.

## Background

Programmed cell death 1 (PD-1), programmed cell death 1 ligand 1 (PD-L1) and cytotoxic T-lymphocyte-associated antigen 4 (CTLA-4) are targets for checkpoint inhibition in cancer immunotherapy [1]. Since the anti-CTLA-4 IgG1 monoclonal antibody ipilimumab was first approved for the treatment of malignant melanoma in 2011, several other immune checkpoint inhibitors have gained approval, namely the PD-1 inhibitors pembrolizumab and nivolumab in 2014, and the PD-L1 inhibitors atezolizumab in 2016, and avelumab and durvalumab in 2017. Despite their clinical efficacy, ICI use carries potential risks for a broad spectrum of unfavorable immune-mediated tissue damage, termed immune-related adverse events (irAEs). The increasing use of ICIs is resulting in accumulating numbers of cases of endocrine irAEs such as thyroid dysfunction, hypophysitis, type 1 diabetes mellitus, and primary or secondary adrenal insufficiency [2, 3]. However, clinical characteristics of secondary adrenal insufficiency and hypophysitis specifically associated with pembrolizumab treatment remain unknown because of the rarity of this adverse event. Here, we present a case of isolated adrenocorticotropic hormone (ACTH) deficiency possibly due to hypophysitis during single-agent pembrolizumab therapy for advanced squamous cell lung cancer.

## Case presentation

An 85-year-old Japanese woman with squamous cell lung carcinoma presented with rapidly worsening general fatigue and appetite loss over two days, eight days after the eighth cycle of pembrolizumab monotherapy. Stage IV squamous cell lung cancer (T3N2M1a) had been diagnosed seven months prior to this. Pembrolizumab had been administered as first-line therapy because biopsy indicated that the PD-L1 tumor proportion score was 100% and no epidermal growth factor receptor mutations, anaplastic lymphoma kinase rearrangements and c-ros oncogene 1 rearrangements were present. The patient had received eight cycles of 200 mg pembrolizumab every three weeks, resulting in stable disease. Routine examination with thyroid hormones and the hypothalamic-pituitary-adrenal axis before each pembrolizumab infusion had shown no significant changes up to the eighth cycle (Table 1). There was no history of concomitant steroid use during immunotherapy, and the patient denied any recent steroid use.

**Table 1:** Time course of laboratory parameters during outpatient care and on arrival

	Before administration	2nd cycle	4th cycle	5th cycle	7th cycle	8th cycle	On arrival		
White blood cell count	5700	6200	6000	5600	5500	6600	6100	/μL	(3300-8600)
Neutrophil	55.7	54.8	63.3	57.9	59.0	59.2	60.2	%	(40–70)
Eosinophil	3.8	4.3	4.8	4.9	3.5	3.1	3.7	%	(0–5)
Sodium	143	142	139	143	142	139	122	mmol/L	(138–145)
Glucose	107	116	99	100	90	102	68	mg/dL	(73–109)
Cortisol	6.67	5.42	8.66	6.23	9.98	7.16	0.92	μg/dL	(6.24–18.0)
ACTH	19.0	NA	NA	NA	NA	NA	8.3	pg/mL	(7.2–63.3)
DHEA-S	NA	NA	NA	NA	NA	NA	5	μg/dL	(7–177)
TSH	2.05	2.13	2.00	2.37	2.45	2.15	3.39	μIU/mL	(0.34–3.8)
FT3	2.77	2.27	2.13	2.1	2.21	2.14	2.65	pg/mL	(2.0–3.8)
FT4	0.83	1.02	0.93	0.84	0.81	0.83	0.86	ng/dL	(0.8–1.5)
Prolactin	NA	NA	NA	NA	NA	NA	18.3	ng/mL	(4.1–28.9)
LH	NA	NA	NA	NA	NA	NA	15.0	mIU/mL	(5.72–64.31)
FSH	NA	NA	NA	NA	NA	NA	50.9	mIU/mL	(< 157.79)
Estradiol	NA	NA	NA	NA	NA	NA	9.3	pg/mL	(< 47.0)
Progesterone	NA	NA	NA	NA	NA	NA	< 0.05	ng/mL	(< 0.33)
GH	NA	NA	NA	NA	NA	NA	3.83	ng/mL	(0.13–9.88)
IGF-1	NA	NA	NA	NA	NA	NA	78	ng/mL	
ADH	NA	NA	NA	NA	NA	NA	3.1	pg/mL	(< 2.8)

Laboratory parameters were obtained before breakfast on the morning before starting administration, and before the second, fourth, fifth, seventh, and eighth cycle of pembrolizmab infusion. Parameters measured on arrival of 1300 h are also shown. Available reference ranges for 85 year-old postmenopausal Japanese women are shown in parentheses. *ACTH* adrenocorticotropic hormone; *ADH* antidiuretic hormone; *DHEA-S* dehydroepiandrosterone sulfate; *FSH* follicle stimulating hormone; *FT3* free triiodothyronine; *FT4* free thyroxine; *GH* growth hormone; *IGF1* insulin-like growth factor 1; *LH* luteinizing hormone; *NA* not applicable; *TSH* thyroid stimulating hormone

On physical examination, body mass index was 20.6 kg/m^2^ (height 153 cm, weight 47 kg), body temperature was 37.0 °C, blood pressure was 112/60 mmHg with a regular pulse of 96 beats/min. The patient was debilitated, but there were no remarkable findings for the head, neck, chest, abdomen or extremities. No visual deficit was apparent according to the confrontation method.

**Fig. 1:**
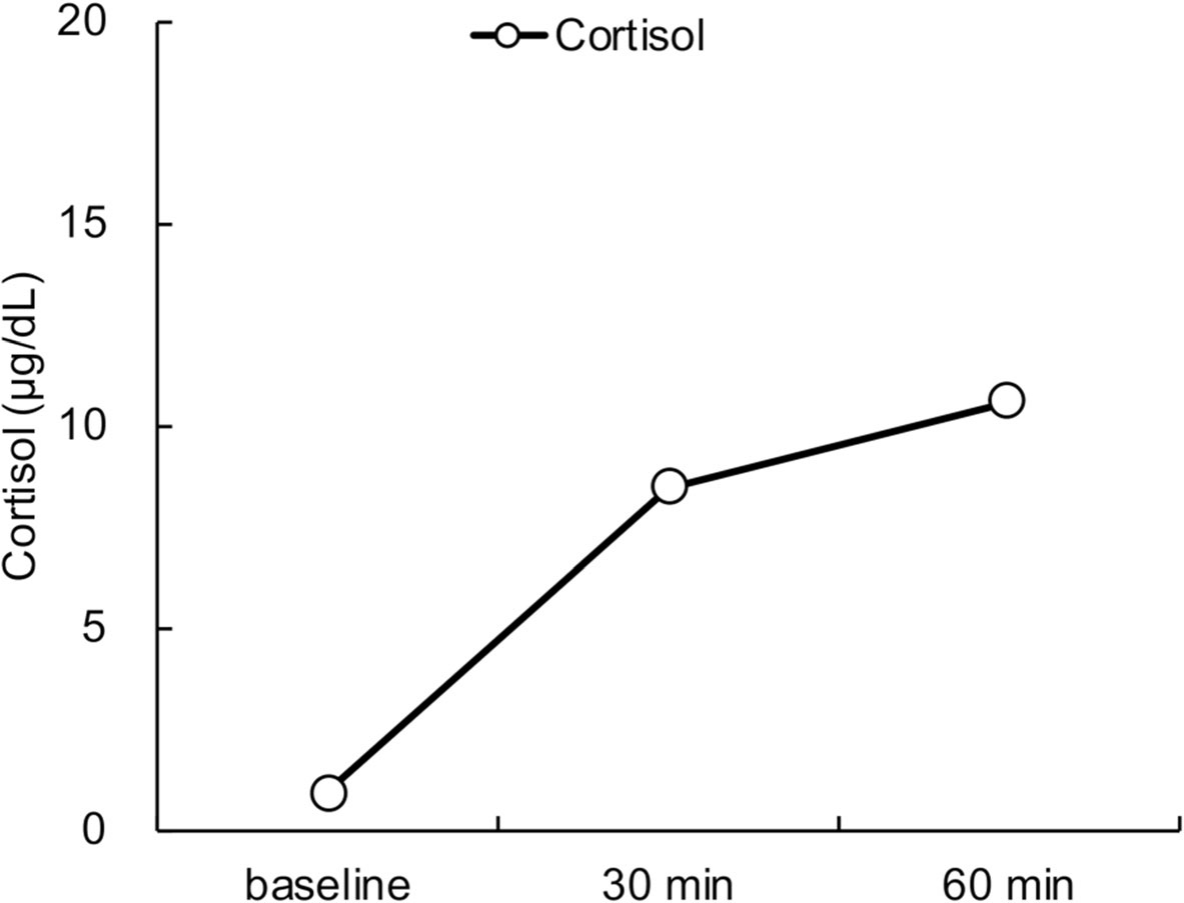
Adrenocorticotropic hormone stimulation test. Horizontal axis: time course. Vertical axis: serum cortisol level

**Fig. 2:**
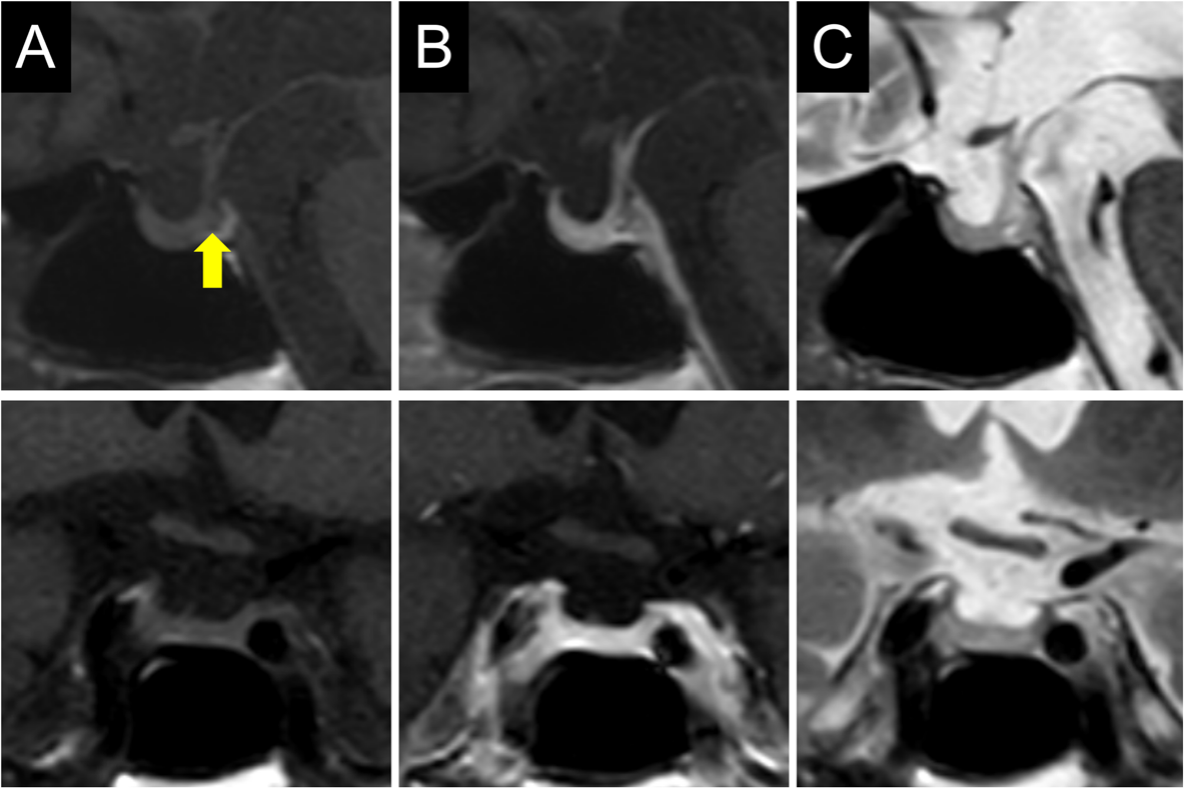
Magnetic resonance imaging of the pituitary. Upper: sagittal plane. Lower: coronal plane. **a** T1-weighted image; Yellow arrow indicates the loss of pituitary bright spot. **b** Gadolinium-enhanced image. **c** T2 weighted image

**Fig. 3:**
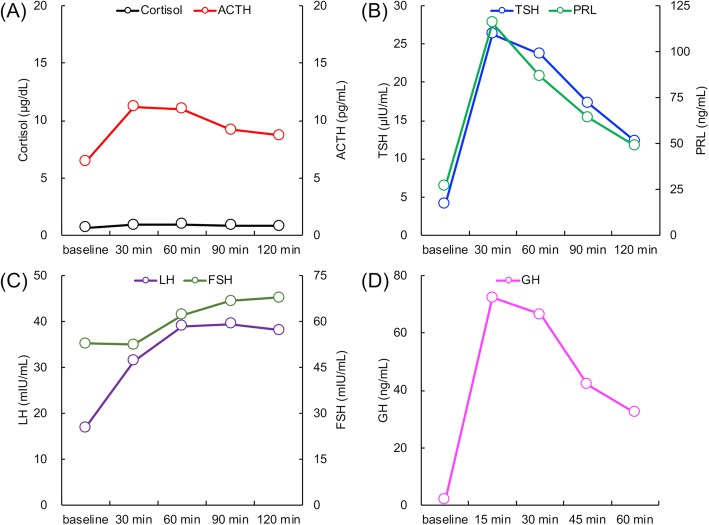
Pituitary challenge tests. Horizontal axis: time course. Vertical axis: hormone levels. **a** Corticotropin-releasing hormone test. **b** Thyrotropin-releasing hormone test. **c** Gonadotropin-releasing hormone test. **d** Growth hormone-releasing peptide 2 test. ACTH, adrenocorticotropic hormone; FSH, follicle stimulating hormone; GH, growth hormone; LH, luteinizing hormone; PRL, prolactin; TSH, thyroid stimulating hormone

Laboratory examination on arrival (1300 h) revealed a low serum cortisol level with an inappropriately low ACTH level, hyponatremia with a raised antidiuretic hormone (ADH) level, and hypoglycemia, but no eosinophilia (Table 1). Urine osmolarity was inappropriately high at 630 mOsm/kg despite low plasma osmolarity of 247 mOsm/kg, indicating inappropriate ADH secretion. There was a poor response to standard-dose short ACTH testing (Fig. 1). Pituitary magnetic resonance imaging revealed higher intensity on T2-weighted imaging, homogeneous gadolinium distribution in the pituitary and the stalk on enhanced imaging, and the lack of a posterior pituitary bright signal on T1-weighted imaging (Fig. 2). However, pituitary enlargement was not apparent. Serum anti-pituitary cell antibody-1 (BML, Inc., Tokyo, Japan) was negative. Urinary free cortisol excretion was 9.8 μg/day (range, 11.2–80.3 μg/day). There were no cortisol responses and poor ACTH (basal at 6.4 to peak at 11.2 pg/mL) secretion responses on corticotropin-releasing hormone testing, and the values for other anterior pituitary hormones on challenge tests were all unremarkable (Fig. 3). The insulin tolerance test was avoided in consideration of the patient’s advanced age. The adrenal glands were unremarkable on computed tomography. Thus, the patient was diagnosed as having isolated ACTH deficiency. Hydrocortisone administered at 15 mg/day, split over three times daily (7.5–5-2.5 mg), swiftly improved the patient’s general condition, corrected the inappropriately high ADH level, and normalized serum sodium concentration. While urine volume increased to about 3000 mL/day after hydrocortisone supplementation, the hypertonic saline test showed a substantial ADH response. Polyuria spontaneously improved with time.

The patient was discharged, and levels of ACTH and cortisol after the morning hydrocortisone, and thyroid function, were followed monthly in outpatient care. While cortisol levels ranged from 8.12 to 18.3 μg/dL, ACTH levels were not detectable on any occasion during the observation period (5 months after admission). Thus, ACTH deficiency obviously persisted, while thyroid function remained normal, and white blood cell count, eosinophil count, serum electrolytes and glucose were all unremarkable.

## Discussion and conclusions

Lung cancer is a leading cause of cancer-related mortality, with the majority of cases being non-small cell lung carcinoma (NSCLC). Immune checkpoint inhibition targeting PD-1 enhances peripheral T-cell immunity, resulting in an antitumor effect, and has shown substantial potential for the treatment of several malignancies including NSCLC [4]. Recently, the anti-PD-1 IgG4 monoclonal antibody pembrolizumab has been recommended as first-line monotherapy for advanced NSCLC with high PD-L1 expression, but lacking targetable driver gene mutations, because of its superiority over conventional platinum-based chemotherapy for both progression-free and overall survival [5, 6]. While ICIs are promising agents for cancer treatment, their use carries a potential risk of irAEs including endocrinopathies (i.e. hypophysitis, hypopituitarism, autoimmune diabetes and thyroiditis). Ten et al. recently reviewed cases of hypophysitis and anterior pituitary insufficiency [7]. Patient median age was 61 years, most were male, median onset from the initiation of immune checkpoint inhibitor use was 12 weeks, and most cases were associated with the use of the CTLA-4 inhibitor ipilimumab [7].

The present case was diagnosed as having ACTH deficiency based on low basal cortisol level, inadequate cortisol secretion responses on a rapid ACTH test and a CRH test, and an insufficient ACTH secretion response on a CRH, although data on insulin tolerance were lacking [8]. Additionally, the low dehydroepiandrosterone sulfate level of 5 ng/mL (reference range for females aged 71 years and above, 7–177 ng/mL) was also compatible with this diagnosis, because patients with ACTH deficiency were reported to have significantly lower dehydroepiandrosterone sulfate than those without [9]. Recently, several cases of isolated ACTH deficiency associated with the use of the PD-1 inhibitor nivolumab have been reported [3]. However, cases associated with pembrolizumab have rarely been described. To the best of our knowledge, at the time of writing there were only two reports of ACTH deficiency associated with pembrolizumab use (Table 2) [10, 11]. These findings suggest a difference in incidence rates of this adverse event even among these drugs in the same class. There are differences between our present case and these previous two reports in the treatment regimen and the onset of pituitary disorders. Whether pembrolizumab or cancer chemotherapy were involved in disease etiology were unclear in the previous reports, but this present case certainly suggests that isolated ACTH deficiency can occur during single-agent pembrolizumab treatment. This present case also suggests the possible occurrence of pituitary damage at early onset, although previous reports showed ACTH deficiency occurred after stopping pembrolizumab.

**Table 2:** Previously reported cases of secondary adrenal insufficiency associated with pembrolizumab treatment

Age	Sex	Disease	Therapeutic agents	Onset	Description of pituitary imaging
60	male	lung cancer	pembrolizumab cisplatin pemetrexed	15 months after stopping pembrolizumab	no pituitary gland enlargement
55	female	breast cancer	pembrolizumab nab-paclitaxel carboplatin adriamycin cyclophosphamide	6 months after stopping pembrolizumab	no pituitary metastasis

The mechanism responsible for the development of ACTH deficiency associated with pembrolizumab remains to be fully elucidated, but hypophysitis is a possible etiology in the present case. Lymphocytic hypophysitis, the most common form of primary hypophysitis, typically gives rise to anterior pituitary dysfunction and marked pituitary enlargement resulting in mass effects [12]. The incidence of such mass effects, resulting in headache or visual impairment, was reported to be as high as 56–83% [12]. In addition to pituitary enlargement, homogeneous strong gadolinium uptake, loss of bright signal intensity in the posterior pituitary lobe and thickened pituitary stalk are signs of lymphocytic hypophysitis on magnetic resonance imaging [13]. In contrast, pituitary enlargement was reported as mild, and thus mass effects such as headache and visual defects are rare in patients with ICI-associated hypophysitis. A case of hypophysitis associated with CTLA-4 blockade was first described in 2003, after which a substantial number of subsequent cases of ipilimumab-induced hypophysitis have been reported due to the increased use of this agent in clinical practice, and a higher incidence of hypophysitis relative to other ICIs [14, 15]. Although the etiology has not been fully elucidated, previous studies suggest that ipilimumab can bind CTLA-4 antigen expressed in the pituitary to evoke lymphocytic infiltration resulting in pituitary toxicity [16, 17]. In patients with ipilimumab-induced hypophysitis, pituitary enlargement and thickened stalk are generally mild or sometimes not present at all [12, 15, 18]. Correspondingly, a recent review reported that none of 57 ipilimumab-induced hypophysitis cases suffered visual impairment [15]. Despite the lower frequency of cases relative to ipilimumab-induced hypophysitis, several instances of hypophysitis associated with the use of the PD-1 inhibitor nivolumab have been reported. These showed a more trivial pituitary enlargement than typical for lymphocytic hypophysitis [3, 19]. Thus, pituitary enlargement on imaging might have too low a sensitivity for diagnosis in such a setting. Diffuse substantial gadolinium enhancement, T2 hyperintensity, and loss of pituitary bright spot on magnetic resonance imaging in this present case might be associated with pituitary inflammation, although pituitary enlargement was not apparent.

Hypophysitis is a well-known endocrine irAE, but its incidence varies depending on the ICI agent employed. A recent systematic review and meta-analysis reported that hypophysitis was rarely observed in patients treated by PD-1 or PD-L1 blockade, despite its more frequent occurrence in patients on combination therapy of CTLA-4 together with PD-1 inhibitors and with CTLA-4 inhibitors alone [2, 20]. Early recognition of hypophysitis and subsequent ACTH deficiency is crucial because it is potentially life-threatening but remediable by steroid replacement therapy. A retrospective cohort study indicated that eosinophilia is an early indicator of ACTH deficiency during PD-1 inhibitor treatment [21]. However, these findings were not applicable to the present case, because the complete blood count did not show any change even at the last cycle of pembrolizumab (Table 1). Because of its rarity, a case of hypophysitis associated with pembrolizumab treatment has yet to be described in the literature. Thus, further cases need to be accumulated to fully comprehend disease characteristics and clinical course.

Conclusively, isolated ACTH deficiency can suddenly occur during pembrolizumab monotherapy in the absence of any warning findings at routine examination. Caution should be exercised for early recognition of adrenal insufficiency so that swift recovery can be achieved by steroid replacement.

## Data Availability

The data used in this current report are available from the corresponding author on reasonable request.

## Abbreviations

ACTH: Adrenocorticotropic hormone
ADH: Antidiuretic hormone
CTLA-4: Cytotoxic T-lymphocyte-associated antigen 4
ICI: Immune checkpoint inhibitor
irAE: Immune-related adverse event
NSCLC: Non-small cell lung carcinoma
PD-1: Programmed cell death 1
PD-L1: Programmed cell death 1 ligand 1
